# CLN6 disease caused by the same mutation originating in Pakistan has varying pathology

**DOI:** 10.1016/j.ejpn.2013.04.011

**Published:** 2013-11

**Authors:** Rita Guerreiro, Jose T. Bras, Mariana Vieira, Varun Warrier, Shakti Agrawal, Helen Stewart, Glenn Anderson, Sara E. Mole

**Affiliations:** a Department of Molecular Neuroscience, Institute of Neurology, University College London, Queen Square, London WC1N 3BG, UK; b MRC Laboratory for Molecular Cell Biology, University College London, Gower Street, London WC1E 6BT, UK; c Division of Biosciences, University College London, Gower Street, London WC1E 6BT, UK; d Birmingham Children's Hospital, Birmingham University, Birmingham Children's Hospital NHS Foundation Trust, Birmingham B4 6NH, UK; e Department of Clinical Genetics, Churchill Hospital, Oxford University NHS Trust, Oxford OX3 7LJ, UK; f Department of Histopathology, Great Ormond Street Hospital for Children, London WC1N 3JH, UK; g UCL Institute of Child Health, 30 Guilford Street, London WC1N 1EH, UK; h Department of Genetics, Evolution and Environment, University College London, Gower Street, London WC1E 6BT, UK

**Keywords:** Batten, Neuronal ceroid lipofuscinosis, NCL, CLN6, Pathology, Prenatal

## Abstract

The neuronal ceroid lipofuscinoses (NCLs), the most common neurodegenerative diseases in children, are characterised by storage of autofluorescent material that has a characteristic ultrastructure. We report two families with variant late infantile NCL, both originating from Pakistan. Probands from both families were homozygous for the same mutation (c.316dupC) but had variable pathology to that currently thought to be typical for CLN6 disease, late infantile variant. The observed pathology of one proband resembled condensed fingerprints, previously described in late infantile CLN7 and CLN8 diseases, and pathology from the second proband was thought to be absent even after repeated skin biopsy, but observed after review. This mutation is the most common NCL mutation in families originating from Pakistan and could be prioritised for testing. Finally, this report contains the first prenatal diagnosis for late infantile CLN6 disease, initially made on the basis of EM and now confirmed by mutation analysis.

## 1 Introduction

The neuronal ceroid lipofuscinoses (NCLs) are the most common neurodegenerative diseases in children, characterised by storage of autofluorescent material that has a characteristic ultrastructure.^1^ Mutations in seven known genes (*CLN2*/*TPP1*, *CLN5*, *CLN6*, *CLN7*/*MFSD8*, *CLN8*, and *CLN1*/*PPT1* or *CLN10*/*CTSD*) cause NCL disease with onset in late infancy.^2^, ^3^ CLN1, CLN2 and CLN10 diseases can be diagnosed by enzyme assay, the remainder can be distinguished only by sequencing to identify or exclude a disease-causing variation. However, the ultrastructural pathology of the storage material is commonly used to guide the order of sequential sequencing.^4^

We report two families, both originating from Pakistan, in which the probands were homozygous for the same mutation but had variable pathology to that currently thought to be typical for CLN6 disease, late infantile variant.

## 2 Case reports

### 2.1 Case 1 (UCL468)

**Fig. 1:**
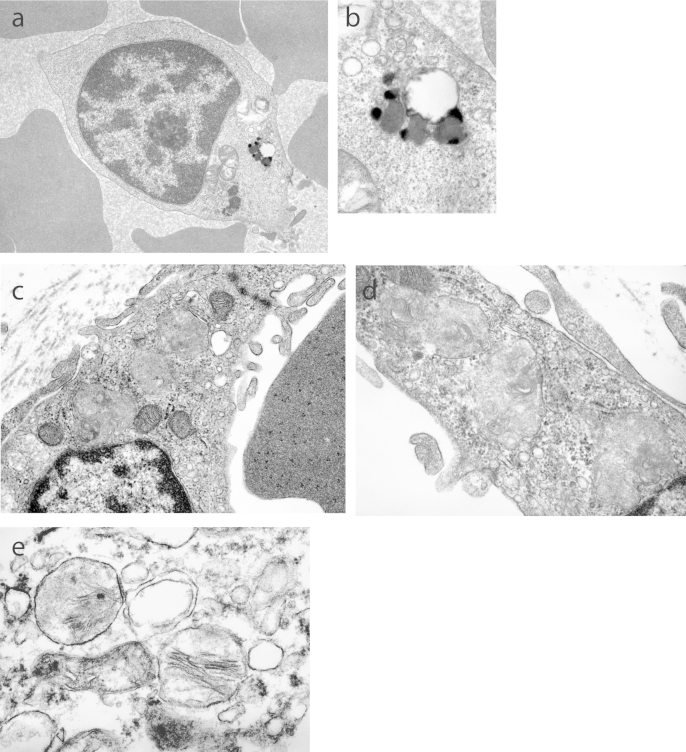
UCL468 proband sample image of: (a) buffy coat lymphocyte with a discrete, membrane bound, lipopigment storage inclusion and (b) high power image demonstrating the fingerprint profile of the storage material. UCL468 sibling foetus chorionic villus sample image of: (c) subtrophoblastic blood vessel endothelial cell with three storage inclusions that are amorphous, and of medium electron density with occasional stacks of lamellae, and (d) fibroblast with three membrane bound inclusions. (e) High power image of inclusions with lamellae identified in various foetal tissues (14 weeks) including the CNS.

The proband was born to parents who were first cousins originating from Pakistan. Normal development was observed until his first seizure just before the age of 3 years after which he became unsteady on his feet, and developed myoclonic epilepsy. There was an 18 month history of nystagmus, ataxia and wide based gait, rough skin with increased pigment and a decelerating head circumference. He presented with bilateral optic atrophy and ERG deterioration by age 4. An EEG showed general bursts of sharp and slow waves and brain MRI displayed deficient inferior vermis and large cysterna magna at 4 years; by 5 years the patient had deteriorated, with abnormal myelination and ex-vacuo dilatation of ventricles thereby suggesting an atrophic process. By this age he was blind, in a wheelchair and unable to perform any skills. Later he became bed-ridden, with gastrostomy, and was stiff and uncomfortable when moved. Hearing was retained. He died aged 11 years. Examination of blood films stained with May-Grundwald giemsa showed no evidence of lymphocyte vacuolation. Rectal biopsy and blood samples were examined comprehensively by light microscopy and subsequently, ultrastructural analysis by transmission electron microscopy (EM). Submucosal ganglion cells showed prominent storage granules and stained positively with periodic acid Schiff's reaction, Luxol fast blue, Sudan black. The storage material exhibited strong autofluorescence when examined by ultraviolet light. The smooth muscle cells of the muscularis layer also had autofluorescent inclusions and stained positively for acid phosphatase. A muscle biopsy showed large active acid phosphatase positive lysosomes. Ultrastructural examination confirmed the presence of sparse storage deposits in lymphocytes. The storage material was membrane-bound as compact lipopigments with fingerprint profiles and amorphous material, confirming NCL disease (Fig. 1(a) and (b)). Variant late infantile NCL was diagnosed at age 5. *CLN7* and *CLN8* genes were excluded as the cause of disease by sequencing.

Following diagnosis of NCL but prior to genetic confirmation, there was a pregnancy terminated at 14 weeks due to the presence of inclusions in chorionic villus sample (CVS) (Fig. 1(c) and (d)), with inclusions confirmed in the aborted foetus (Fig. 1(e)). These were amorphous and of low density. A subsequent pregnancy for which CVS was normal resulted in a healthy sister.

DNA from the proband was exome sequenced and whole genome genotyped. Genomic DNA was prepared according to Illumina's TruSeq Sample preparation and Exome Enrichment protocols (Illumina, CA). Captured DNA was sequenced on an Illumina HiSeq 2000 using 2×100 bp paired-end reads. Resulting sequences were aligned to the human genome reference (hg19) and variants called using GATK.^5^ Variants were annotated according to their presence in publicly available databases (1000Genomes and dbSNP build 135). The initial filtering process excluded all synonymous and heterozygous variants present in the proband. There were 57 homozygous variants, of which one was a pathogenic mutation in *CLN6* (NM_017882: c.316dupC: p.Arg106ProfsX26). Both parents were carriers, and DNA from the aborted foetus was homozygous for this same change.

### 2.2 Case 2 (UC593)

The proband is the only child of first degree consanguineous parents from Rawalpindi in northern Pakistan. Early development was normal. His speech and walking began to deteriorate when 3 years old. When 4 years he developed shaking of both legs which progressed to myoclonic jerks within a year, and he experienced his first generalised tonic clonic seizure at age 6, his current age. He now has continuous wringing hand movements, can no longer bear weight and has lost eating and drinking skills. MRI showed cerebral and cerebellar atrophy with ex-vacuo dilation of lateral ventricles. The periventricular white matter and posterior limb of internal capsules along with deep cerebellar white matter showed high T2 signal with hazy appearances. The optic nerve and chiasm were thinner than normal. Flash electroretinograms were absent bilaterally with giant flash visual evoked responses. This was further confirmed with abnormal somatosensory evoked potentials (giant cortical response) and present C reflexes. Giant flash visual responses were evoked at slow flash rate of 1.2 Oz-Fz.

**Fig. 2:**
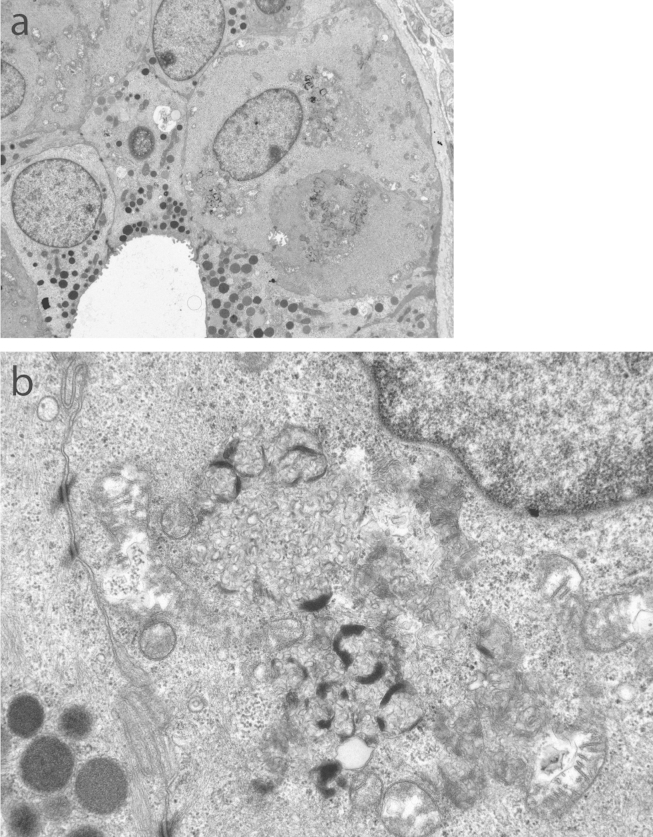
UCL 593 proband sample image of: (a) skin biopsy showing sweat gland epithelial cells with storage material, and (b) high power image demonstrating distinct storage inclusions of mixed type (curvilinear-like and fingerprint profiles).

NCL was suspected. However, ultrastructural examination of a skin biopsy twice failed to reveal any inclusions. EM of buffy coat was not requested. Nevertheless, DNA was submitted for sequencing of genes that typically cause NCL with onset in late infancy and a known pathogenic mutation (c.316dupC: p.Arg106ProfsX26) was found in homozygous form in *CLN6*. Subsequent ultrastructural analysis re-examination of the second skin biopsy by transmission electron microscopy showed distinct storage inclusions in sweat gland epithelium, endothelial cells and smooth muscle cells (Fig. 2). The storage was of a mixed type with curvilinear-like and fingerprint profiles.

### 2.3 Ethical standards

All work was approved by UCL Research Ethics Committee. The parents of the probands gave informed consent prior to inclusion in this study and to its publication.

## 3 Discussion

These two cases demonstrate important considerations regarding pathology in CLN6 disease, and perhaps NCL in general, since both are atypical. In family UCL468 the pathology is more condensed than previously reported for late infantile CLN6 disease, but resembles that reported for CLN7 and CLN8 disease. In contrast, in family UCL593, no pathology was detected to begin with in a skin biopsy which would normally be taken to exclude NCL, however subsequent re-examination of a second biopsy in a different laboratory did reveal pathology that was typical for NCL but was different to that in family UCL468. The pathology in other families carrying the same mutation has not previously been reported in detail, but was mixed curvilinear and fingerprint profiles in endothelial cells and smooth muscle cells of a skin biopsy in families homozygous for the same mutations (UCL423 and UCL233) and mixed curvilinear and fingerprint profiles in conjunctival biopsy from a family heterozygous for the same mutation (UCL202) (unpublished data). Thus, pathology for the group of variant late infantile NCLs may vary and cannot be reliably used as a guide to order of gene analysis. In addition, and importantly, NCL gene analysis should still be considered in a child showing typical clinical symptoms even if pathology has been reported as absent.

There are now five Pakistani family described with this mutation in *CLN6*,^6^ suggesting a founder mutation that is predicted to introduce a premature stop codon. In one family homozygous for the same mutation (UCL423) the mutant protein was no longer detected by polyclonal antisera to CLN6.^7^

Other Pakistani families diagnosed with NCL carry private mutations in *CLN5* (two mutations), *CLN6* (one additional mutation to that reported here), *CLN8* (three mutations) and *CLN10* (two mutations), with none yet reported causing CLN1, CLN2, CLN3, CLN4 or CLN7 diseases.^2^, ^8^ Thus, it should be high priority to test for this *CLN6* mutation in Pakistani families with any type of suspected NCL, but particularly with onset in late infancy, regardless of ultrastructural pathology of storage material, or even its apparent absence. Mutations in *CLN6* are now known to cause disease whose age of onset ranges from late infancy to adult.^9^ This report demonstrates the power of second generation sequencing technologies to very quickly identify the causative gene in families which are atypical or where the mostly likely candidate genes have been excluded. Given the challenge experienced here of observing pathology in a skin biopsy, electron microscopy on buffy coat could be considered earlier in the diagnostic pathway.

In conclusion, NCL should be considered in children with a consistent clinical picture and normal levels of PPT1, TPP1 or CTSD, even in the absence of obvious inclusions on skin biopsy, and a biopsy should be reviewed when the suspicion of NCL remains high. The choice of gene testing should be influenced more by the ethnic background of the affected child than the pattern of ultrastructural deposits.

Finally, it is worth noting that this is the first report of prenatal diagnosis for CLN6 disease late infantile variant, initially made on the basis of electron microscopy (EM) and now confirmed by mutation analysis. Caution should be used in applying EM to prenatal diagnosis – direct detection of the causative mutation(s) is preferable. Interestingly, in this one family, the inclusions detected in the CVS and confirmed in the foetus did not have the fingerprint profile and condensed nature of those in the proband but were more amorphous and of less density suggesting that these inclusions are in the very early stages of formation.
